# Adaptive Cross-Modal Denoising: Enhancing LiDAR–Camera Fusion Perception in Adverse Circumstances

**DOI:** 10.3390/s26020408

**Published:** 2026-01-08

**Authors:** Muhammad Arslan Ghaffar, Kangshuai Zhang, Nuo Pan, Lei Peng

**Affiliations:** 1 Shenzhen Institutes of Advanced Technology, Chinese Academy of Sciences, Shenzhen 518055, China; ghaffar@siat.ac.cn (M.A.G.); ks.zhang@siat.ac.cn (K.Z.); nuo.pan@siat.ac.cn (N.P.); 2 University of Chinese Academy of Sciences, Beijing 100049, China

**Keywords:** multimodal perception, LiDAR–camera fusion, semantic-guided restoration, autonomous driving, attention mechanism, noise removal, real-time sensor enhancement

## Abstract

**Highlights:**

**What are the main findings?**

- An Adaptive Cross-Modal Denoising (ACMD) framework is presented, introducing a reliability-driven uni-directional fusion mechanism that selectively refines the noisy modality using semantic cues from the cleaner sensor.
- A novel attention-based ABC + CMD pipeline is developed, enabling efficient noise-aware feature alignment and outperforming state-of-the-art unimodal and multimodal denoising methods across LiDAR–camera perception tasks.

**What are the implications of the main findings?**

- ACMD enhances the robustness of autonomous perception in adverse weather by achieving large gains in PSNR, Chamfer Distance, and Joint Denoising Effect, without adding computational burden.
- The plug-and-play ACMD design generalizes to any encoder–decoder backbone, making it suitable for deployment in real-time AV systems and for future multimodal sensing combinations (LiDAR–thermal, radar–camera).

**Abstract:**

Autonomous vehicles (AVs) rely on LiDAR and camera sensors to perceive their environment. However, adverse weather conditions, such as rain, snow, and fog, negatively affect these sensors, reducing their reliability by introducing unwanted noise. Effective denoising of multimodal sensor data is crucial for safe and reliable AV operation in such circumstances. Existing denoising methods primarily focus on unimodal approaches, addressing noise in individual modalities without fully leveraging the complementary nature of LiDAR and camera data. To enhance multimodal perception in adverse weather, we propose a novel Adaptive Cross-Modal Denoising (ACMD) framework, which leverages modality-specific self-denoising encoders, followed by an Adaptive Bridge Controller (ABC) to evaluate residual noise and guide the direction of cross-modal denoising. Following this, the Cross-Modal Denoising (CMD) module is introduced, which selectively refines the noisier modality using semantic guidance from the cleaner modality. Synthetic noise was added to both sensors’ data during training to simulate real-world noisy conditions. Experiments on the WeatherKITTI dataset show that ACMD surpasses traditional unimodal denoising methods (Restormer, PathNet, BM3D, PointCleanNet) by 28.2% in PSNR and 33.3% in CD, and outperforms state-of-the-art fusion models by 16.2% in JDE. The ACMD framework enhances AV reliability in adverse weather conditions, supporting safe autonomous driving.

## 1. Introduction

**Figure 1:**
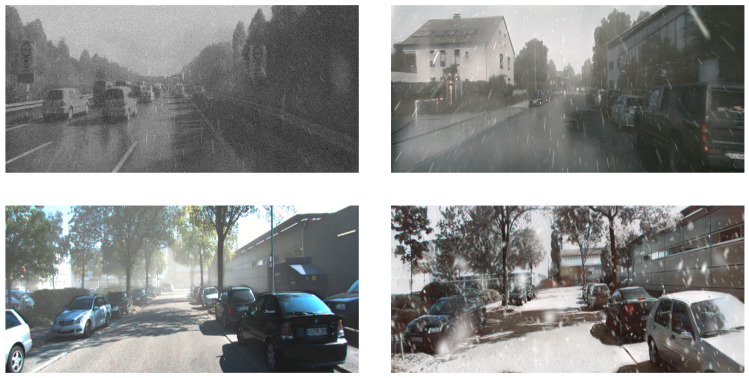
Critical weather impact on camera data.

Autonomous Vehicles (AVs) require precise and reliable sensor data to navigate and perceive their environments effectively. LiDAR and camera systems form the foundation of modern perception pipelines by providing complementary 3D geometric and visual information. However, their reliability is often compromised under adverse weather conditions such as fog, rain, snow, and low-light environments. Studies report that up to 70% of AV perception failures in field trials are directly linked to sensor degradation in challenging weather [1], making this a critical bottleneck for large-scale AV deployment. LiDAR suffers from scattering effects, which cause depth measurement inaccuracies and sparse returns, while cameras face reduced visibility, motion blur, and incomplete scene information. These degradations manifest in missed detections and false positives (often producing ghost objects), which in turn lead to unsafe driving behaviors such as unnecessary braking or dangerous evasive maneuvers [2]. Addressing this challenge is therefore fundamental to ensuring the safe and efficient operation of AVs across diverse environmental conditions. Figure 1 illustrates representative camera degradations under adverse weather conditions.

Early efforts to mitigate weather-induced degradation primarily relied on weather-specific simulation-based training strategies [3]. Although effective in controlled scenarios, these methods require costly retraining for each weather condition, often degrade performance under normal conditions, and generalize poorly to the complex and continuously varying environments encountered in real-world driving [4]. Traditional denoising approaches, which are typically designed for a single modality, suppress noise locally but fail to recover missing or corrupted semantic information. As a result, they cannot fully exploit the complementary nature of multimodal perception systems, where different sensors provide overlapping yet distinct information [5].

Recent advances in multimodal perception have sought to fuse LiDAR and camera features to improve robustness and scene understanding. Most existing fusion frameworks adopt symmetric cross-attention or bidirectional feature exchange, treating both modalities as equally reliable at all times [6]. While effective under balanced sensing conditions, such designs become problematic under asymmetric degradation, a common occurrence in adverse weather. For example, dense fog may severely corrupt LiDAR geometry while leaving camera textures relatively intact, whereas nighttime or motion blur may degrade visual signals while LiDAR remains reliable. Symmetric or bidirectional fusion in these cases risks propagating noise from the degraded modality into the cleaner one, leading to feature contamination, increased computational cost, and reduced interpretability [7]. A more integrated approach that considers both sensor modalities together is crucial for achieving optimal denoising performance and improving the overall system’s robustness. These limitations highlight the need for a real-time approach that can effectively combine the strengths of both modalities to enhance sensor data quality and perception accuracy.

To address these limitations, this study introduces an Adaptive Cross-Modal Denoising (ACMD) framework that explicitly departs from symmetric fusion paradigms. ACMD is designed around the principle of reliability-aware, unidirectional semantic transfer, where information flows only from the cleaner modality to the noisier one. The framework operates through a sequence of structured stages. First, each modality undergoes independent self-denoising to suppress modality-specific noise. Second, the Adaptive Bridge Controller (ABC) evaluates the residual reliability of the self-denoised encoder features and determines the dominant (cleaner) modality. Finally, Cross-Modal Denoising (CMD) performs targeted refinement exclusively on the degraded modality, guided by semantic information from the reliable sensor. By avoiding redundant bidirectional fusion, ACMD prevents noise amplification, reduces unnecessary computation, and preserves the intrinsic strengths of each sensing modality.

The effectiveness of the ACMD framework is validated on the WeatherKITTI dataset, where it demonstrates substantial improvements in multimodal perception robustness. Compared to existing fusion-based methods such as GAFusion and BEVFusion4D, ACMD achieves a 16.2% improvement in Joint Denoising Effect (JDE) while maintaining real-time performance. These results highlight the advantages of reliability-aware, unidirectional cross-modal denoising and establish ACMD as a practical and robust solution for autonomous driving in adverse weather conditions.

## 2. Related Work

### 2.1. Denoising in LiDAR and Camera Data

Recent advancements in 3D object detection have underscored the importance of sensor denoising in enhancing detection accuracy. The quality of input data is crucial for object detection, and removing noise from sensor modalities, especially LiDAR and camera data, is essential. However, most research has focused on denoising individual modalities, often overlooking the benefits of utilizing the complementary nature of both LiDAR and camera data. Zhi et al. [8] introduced DefDeN, a 3D object detection framework that addresses the perception challenges faced by autonomous driving systems. DefDeN leverages attention mechanisms and gated information networks to integrate features from both LiDAR and camera data. To overcome issues such as slow convergence, high computational costs, and increased false positives, the model incorporates deformable attention mechanisms, noise addition and denoising modules, and contrastive learning strategies.

Hu et al. [9] proposed a denoising method for LiDAR return signals using the CAENN approach, which applies an encoding-decoding structure with convolutional neural networks (CNNs) to extract deep features from LiDAR return signals. This method reduces noise while preserving relevant signals. Zhang et al. [10] introduced a real-time point cloud denoising model based on improvements to the PP-LiteSeg framework. By enhancing the feature extraction and fusion modules, this model significantly improves segmentation and denoising accuracy, handling noise from rain and fog without sacrificing inference speed. The model achieved an 11.1% improvement in mean Intersection over Union (mIoU) and maintained real-time inference at 205.06 FPS. Zhao et al. [11] proposed TripleMixer, a point cloud denoising model that integrates spatial geometric features, frequency domain features, and multi-channel contextual information, achieving excellent denoising performance on both real-world and newly proposed datasets.

### 2.2. Sensor Fusion Techniques

Sensor fusion methods, such as BEVFusion4D [12], GAFusion [13], and MSMDFusion [14], have been widely explored for enhancing object detection by combining LiDAR and camera data. However, these methods rely heavily on rigid sensor alignment and projection matrices, limiting their flexibility in dynamic environments. LiDAR-based object detection methods are typically categorized into direct point cloud processing, voxel-based methods, and multi-view projection methods [15]. The direct point cloud processing method, introduced by Qi et al. in PointNet [16], utilizes neural networks to extract features directly from LiDAR point clouds. However, PointNet struggles with local feature extraction, prompting the development of PointNet++ [17], which uses a hierarchical network structure for better feature extraction at various scales. PointRCNN [18] further improved this by introducing local and global feature learning layers, enhancing detection accuracy in complex scenes. Although these methods offer high accuracy, they require significant computational resources when processing large point clouds.

To address computational inefficiency, voxel-based methods, such as VoxelNet [19] were introduced. VoxelNet converts point clouds into fixed-size 3D units, simplifying data representation. However, voxelization often results in sparse data and inefficiency. PointsPool, introduced by Yang et al. in STD [20], improves this by transforming sparse features into compact representations for more efficient detection. Despite this, voxel-based methods still consume substantial computational resources. Data fusion techniques, such as BEVDetNet [21] and FVNet [22], project point clouds onto 2D Bird’s Eye View (BEV) or cylindrical surfaces to reduce computational complexity. However, such projection methods can lead to information loss, especially when detecting objects from a single view, resulting in missed or false detections.

## 3. Adaptive Cross-Modal Denoising

### 3.1. Framework

**Figure 2:**
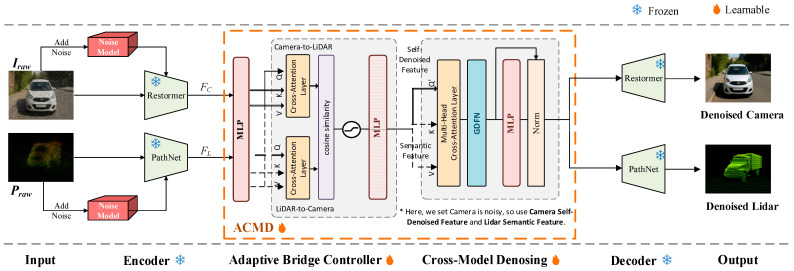
Architecture of the proposed Adaptive Cross-modal Denoising (ACMD) framework. Note *: In this example, the camera modality is degraded; therefore, camera features are used as queries, while LiDAR features serve as keys and values and are forwarded to the cross-modal denoising module to guide the restoration process.

The proposed Adaptive Cross-Modal Denoising (ACMD) framework is designed as a plug-in module inserted between the encoder and decoder of a modality-specific denoising network. ACMD introduces two key innovations: the Adaptive Bridge Controller (ABC) and the Cross-Modal Denoising Module (CMD), which together enable reliable, real-time semantic correction between LiDAR and camera streams under adverse weather conditions like fog, rain, and snow. Unlike conventional multimodal pipelines [12,13,14] that fuse modalities symmetrically or apply naive cross-attention, ACMD performs directional, noise-aware refinement, where only the noisier modality is corrected, guided by semantic cues from the cleaner modality. ABC determines the direction, and CMD performs a multi-head cross-attention-based correction, eliminating the need for duplicated fusion branches. The overall architecture of the ACMD framework is illustrated in Figure 2.

Given a multimodal dataset such as WeatherKITTI, each sample consists of a LiDAR point cloud ($P_{raw}$) and a corresponding camera image ($I_{raw}$), the ACMD model (Ψ) aims to reconstruct their denoised forms $\left(\tilde{P},\tilde{I}\right)$. The encoders remove modality-specific noise such as LiDAR scattering and camera motion blur, generating partially denoised feature tensors ($F_{C}$, $F_{L}$) and semantic maps ($S_{C}$, $S_{L}$) for each modality. ABC evaluates residual noise using metrics like point-density variance, intensity entropy, and texture sharpness, deriving reliability scores ($R_{L}$ for LiDAR, $R_{C}$ for camera). Based on these scores, ABC routes semantic guidance from the cleaner modality to the noisier one. The cleaner modality’s semantic tensor is projected to a shared space and fused with the noisy modality’s latent features using gated cross-attention, ensuring unidirectional semantic refinement.

The overall ACMD process can be formally represented as follows:

(1) $$(\tilde{P},\tilde{I})=\Psi \left(\begin{array}{c} \hat{P}(P_{raw}),\hat{I}(I_{raw})\\ \alpha_{C}X_{C}(\hat{P},S_{L}),\alpha_{L}X_{L}(\hat{I},S_{C}) \end{array}\right)$$

where $\alpha_{L}$ and $\alpha_{C}$ are adaptive reliability weights, and $X_{L}$ and $X_{C}$ are cross-modal fusion functions. The CMD’s cleaned output for the degraded modality is sent to its own decoder for reconstruction, while the clean modality bypasses CMD and decodes directly.

As ACMD operates on self-denoised latent features produced by modality-specific encoders, ACMD is compatible with any encoder–decoder architecture. The ACMD framework efficiently adapts to varying noise conditions, using the strengths of both modalities to ensure stable perception, making it suitable for autonomous vehicles in challenging environments. The overall ACMD pipeline consists of three stages:

The overall ACMD pipeline follows a three-stage structure:

Modality-specific encoders transform raw LiDAR and camera inputs into self-denoised latent tensors and mid-level semantic descriptors.

The ACMD operates directly on these encoder outputs: the Adaptive Bridge Controller (ABC) estimates reliability asymmetry between modalities and selects the semantic guidance direction, while the CMD module performs targeted, noise-aware cross-modal refinement on the degraded latent stream.

Modality-specific decoders then reconstruct the final denoised LiDAR point cloud and camera image, with the cleaner modality decoded directly from its self-denoised latent tensor and the noisier modality decoded from the CMD-refined representation.

The detailed descriptions of these components are provided in the subsequent sections.

### 3.2. Encoders

The ACMD framework begins with modality-specific encoders that generate robust latent representations from LiDAR and camera data. These encoders act purely as feature extractors, and their decoder counterparts reconstruct the final denoised outputs. The ACMD’s design does not depend on any specific backbone; it only requires that each modality provides a self-denoised latent representation suitable for cross-modal processing. This ensures ACMD’s compatibility with a broad class of encoder–decoder architectures commonly used in image and point-cloud restoration.

LiDAR and camera exhibit fundamentally different noise behaviors, LiDAR suffers from scattering and sparse returns, whereas camera images degrade through blur, darkening, and color distortion. Attempting cross-modal fusion on raw degraded signals would lead to ambiguous correspondences and unreliable semantic propagation. Before any cross-modal interaction, each modality must first mitigate its own intrinsic degradation, thereby producing semantically meaningful latent tensors suitable for cross-modal reasoning. Therefore, in this work, we adopt Restormer [23] for images and PathNet [24] for point clouds as prototype encoders to generate stable latent features.

Given raw sensor inputs $I_{raw}$ (camera) and $P_{raw}$ (LiDAR), the encoders extract:

(2) $$F_{C}=E_{C}(I_{raw}),\;\;\;\;F_{L}=E_{L}(P_{raw})$$

from intermediate layers, we additionally extract semantic descriptors:

(3) $$S_{C}=\Phi_{C}\left(F_{C}\right),\;\;\;\;S_{L}=\Phi_{L}\left(F_{L}\right)$$

where $F_{C}$ and $F_{L}$ are the modality-specific clean latent tensors, $S_{C}$ and $S_{L}$ are the mid-level semantic descriptors for camera and LiDAR, respectively, $\Phi_{C}$ is the projection operator that captures camera mid-level semantic information (texture, object boundaries, edges), and $\Phi_{L}$ is the shallow feed-forward mapping that extracts LiDAR mid-level semantic information guiding LiDAR restoration.

### 3.3. Adaptive Bridge Controller (ABC)

The Adaptive Bridge Controller is the core decision-making unit of the ACMD framework. Its purpose is to (1) quantify the relative reliability of each modality after independent self-denoising and (2) determine the direction of semantic transfer for CMD. This ensures that information always flows from the cleaner modality to the noisier modality, preventing noise reinforcement and unnecessary computation. Environmental degradation is inherently asymmetric: scattering severely corrupts LiDAR returns but minimally affects camera textures; conversely, low-light or motion blur collapses image details while leaving LiDAR geometry intact. Therefore, multimodal denoising must dynamically adjust the fusion direction based on instantaneous sensor reliability, rather than relying on static heuristics. ABC replaces conventional symmetric fusion with a targeted, unidirectional mechanism that mitigates the propagation of degradation across modalities.

After the self-denoising stage, the ABC considers three reliability conditions. Specifically, these include (1) both modalities remain clean, (2) only one modality is degraded, and (3) both modalities are severely degraded. When both sensors are assessed as clean, their residual noise levels fall below a predefined threshold, the cross-modal denoising process is bypassed, and the features are directly forwarded to downstream tasks, thereby avoiding unnecessary computation. The main focus of this work lies in scenarios where one modality is more degraded than the other. In such cases, ABC identifies the relatively cleaner modality and uses its semantic information to guide the denoising of the noisier modality. In contrast, when both modalities are severely degraded, their latent features may become unreliable, and similarity-based reliability estimation becomes ineffective. Addressing this scenario falls beyond the scope of the present work and is identified as an important direction for future research.

ABC evaluates sensor reliability by measuring semantic coherence in the latent feature space rather than attempting to estimate absolute noise magnitude. Specifically, reliability is quantified based on the alignment between a modality’s self-denoised features and its corresponding cross-attended representation from the other modality. This design reflects the observation that reliable sensor features preserve semantic structure and consistency under noise, while corrupted features exhibit misalignment and instability. We implement ABC as the procedure in Algorithm 1.

ABC receives as input the self-denoised latent tensors ($F_{C}$, $F_{L}$) and their corresponding semantic descriptors ($S_{C}$, $S_{L}$) produced by the encoders. These tensors are concatenated and projected into a shared latent space:

(4) $$H_{C}=W[F_{C}\|S_{C}],\;\;\;\;H_{L}=W[F_{L}\|S_{L}]$$

where [⋅‖⋅] denotes concatenation, and $W\in R^{d\times 2d}$ is a learnable projection matrix.

To evaluate semantic coherence across modalities, ABC applies customized bi-directional attention. Two attention heads operate in parallel:

(5) $$A_{L\to C}=Softmax\left(\frac{(H_{L}W_{Q})(H_{C}W_{K}{)}^{T}}{\sqrt{d_{k}}}\right)H_{C}W_{V},\;\;A_{C\to L}=Softmax\left(\frac{(H_{C}W_{Q})(H_{L}W_{K}{)}^{T}}{\sqrt{d_{k}}}\right)H_{L}W_{V}$$

which represent how well modality X explains modality Y in a semantic sense.

Reliability for each modality is evaluated based on cosine similarity between the modality’s self-denoised features and its cross-attended representation:

(6) $$R_{L}=\cos(H_{L},A_{C\to L}),\;\;\;\;R_{C}=\cos(H_{C},A_{L\to C})$$

where a larger $R_{L}$ or $R_{C}$ implies that the modality exhibits high semantic integrity and, therefore, is less degraded.

Cosine similarity is selected because it measures directional agreement in feature space, making it invariant to feature magnitude and robust to scale variations introduced by different backbones, normalization layers, or activation dynamics. Importantly, it reflects semantic stability rather than raw signal strength: as noise severity increases, feature representations become less structured and exhibit lower alignment with cross-modal semantics, leading to a measurable decrease in cosine similarity. Empirically, we observe a consistent correlation between increased noise and reduced cosine similarity, validating its suitability as a reliability indicator.

The relative reliability difference is then calculated as:

(7) $$\Delta R=|R_{L}-R_{C}|$$

and this difference determines which modality has the stronger semantic consistency and thus, should act as the guide.

After that, a gating function computes the probability of LiDAR being the cleaner modality:

(8) g=σ(M(ΔR))

where M is an MLP and σ is the sigmoid activation. The output (g) directs the flow of information between modalities:

Thus, ABC selects the direction:

(9) $$Guide=\left\{\begin{array}{ll} L\to C, & g>\tau \\ C\to L, & g\leq \tau \end{array}\right.$$

If g>τ, LiDAR guides the camera denoising.

If g≤τ, the camera guides the LiDAR cleaning.

Cross-attention is adopted within ABC to capture global semantic correlations between heterogeneous domains, namely 2D image textures and 3D LiDAR geometry, enabling the controller to reason beyond strictly local neighborhoods and align structures across modalities [25]. The gating mechanism is introduced to map the reliability difference to a continuous guidance weight rather than a hard decision, which avoids abrupt switching between modalities and supports smooth, gradual adaptation as the relative noise conditions of LiDAR and camera evolve over time.

The ABC module enables adaptive, localized semantic refinement by selecting the most appropriate modality to guide the denoising process. This ensures that the reliable modality consistently drives the semantic recovery of the noisy modality, enhancing real-time stability and computational efficiency in the ACMD framework.
**Algorithm 1:** Adaptive Bridge Controller (ABC)[1]**Input:** Self-denoised tensors ($F_{m}$), Mid-level semantic tensors ($S_{m}$)m ∈ {L, C }[2]**Output:** Semantic guidance for CMD*//Flow from clean sensor to noisier sensor*1:**begin** ABC module
2:**for** each input do
3:**Common latent space**
4:$H_{m}=W[F_{m}\|S_{m}]$$m\;\in \;\left\{L,\;C\;\right\}$5:**Bidirectional Cross-attention***//Parallel operation*6:$A_{L\to C}=Softmax\left(\frac{(H_{L}W_{Q})(H_{C}W_{K}{)}^{T}}{\sqrt{d_{k}}}\right)H_{C}W_{V}$
7:$A_{C\to L}=Softmax\left(\frac{(H_{C}W_{Q})(H_{L}W_{K}{)}^{T}}{\sqrt{d_{k}}}\right)H_{L}W_{V}$
8:**Calculate reliability scores for both modalities**
9:$\;\;\;\;\;\;\;R_{L}=\;\cos(H_{L},\;A_{C\to L})$*//Reliability for LiDAR*10:$\;\;\;\;\;\;\;R_{C}=\cos(H_{C},{\;A}_{L\to C})$*//Reliability for Camera*11:$\;\;\;\;\;\;\;\Delta R=|R_{L}-R_{C}|$*//Reliability difference*12:       g=σ(M(ΔR))*//gating function*13:      **if** g > τ **then***//LiDAR is more reliable*14:      guidance mode ← “LiDAR to Camera”*//LiDAR guides Camera*15:       semantic tensor ← $S_{L}$
*//LiDAR semantic tensor as cleane*16:      noisy tensor ← $F_{C}$
*//Camera as noisy modality*17:   **else**

18:           guidance mode ← “Camera_to_LiDAR”*//If Camera is more reliable*19:       semantic tensor ← $S_{C}$
*//Use Camera semantic tensor*20:            noisy tensor ← $F_{L}$
*//LiDAR is noisy*21:   **end if**

22:      **The guidance (semantic flow decision)**

23:      $Guide=\left\{\begin{array}{ll} L\to C, & g>\tau \\ C\to L, & g\leq \tau \end{array}\right.$

24:      return guidance
25:**end for**
26:**End**


### 3.4. Cross-Modal Denoising (CMD)

The Cross-Modal Denoising (CMD) module performs the core semantic refinement in the ACMD pipeline. Unlike conventional fusion strategies [26], which typically merge multimodal features indiscriminately, CMD introduces an adaptive, noise-aware, and unidirectional mechanism. This approach strictly ensures that only the degraded modality is refined without contaminating the clean signal. The goal of CMD is to maintain semantic coherence between the modalities, which is essential for robust multimodal perception in adverse weather or low-light conditions.

After the ABC determines the reliability scores for LiDAR and camera, it provides a reliability decision that defines which sensor acts as the semantic guide and which requires further correction. CMD then receives two inputs: the self-denoised latent feature tensor of the noisier modality ($F_{noisy}$), and the mid-level semantic tensor of the cleaner modality ($S_{clean}$). These tensors are projected into a shared 256-dimensional latent space through lightweight linear mappings to guarantee spatial and feature-scale compatibility:

(10) $$\tilde{F}=F_{noisy}W_{F},\;\;\;\;\tilde{S}=S_{clean}W_{S}$$

and to prevent unreliable features from dominating fusion, CMD introduces a noise-aware variant of attention. Unlike the standard attention mechanism, which uniformly scales queries by $1/\sqrt{d}$, CMD additionally incorporates a reliability-dependent noise factor $\hat{\sigma }$ predicted by ABC:

(11) $$Q^{\prime }=\frac{\tilde{F}W_{Q}}{\sqrt{d}\;(1+\hat{\sigma })}$$

where $\tilde{F}W_{Q}$ is the queries from standard attention, $\sqrt{d}$ is the transformer scaling, and $\hat{\sigma }$ is noise severity (predicted by ABC) controlling the influence of noisy modality. This reduces spurious attention activations from noisy inputs.

The targeted enhanced cross-attention is then calculated as:

(12) $$A=Softmax\left(Q^{\prime }K^{T}\right)V$$

where $K=\tilde{S}W_{K}$ and $V=\tilde{S}W_{V}$ are the key and value matrices from the clean modality. We further introduce a semantic gating mask (G) to constrain attention to reliable-modality’s clean regions:

(13) $$G=\sigma (W_{g}\tilde{S}\;)$$

(14) $$\hat{F}=A⊙G$$

where G amplifies salient clean regions, σ is the sigmoid activation, and $W_{g}$ is a learnable linear layer. ⊙ enforces semantic gating as shown in Equation (14). This mask ensures that refinement is applied selectively, only where the noisy modality exhibits significant degradation, while protecting structurally consistent regions from over-correction.

The noisy features are then updated residually, preserving important features while refining others:

(15) $$F^{*}=\tilde{F}+\hat{F}$$

**Figure 3:**
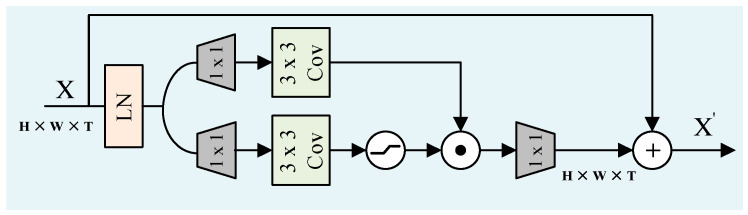
Gated Depthwise Feed-Forward Network (GDFN).

Instead of FFN in the standard attention mechanism, we employed a compact Gated Depthwise Feed-Forward Network (GDFN) with GELU activation (ϕ) and layer normalization for further refinement, as shown in Figure 3. The GDFN selectively controls which features to enhance or suppress based on their relevance to the task, helping refine the features for accurate reconstruction:

(16) $$G=\phi \left(W_{1}^{\left(d\right)}W_{1}^{\left(p\right)}{\tilde{F}}_{noisy}\right)⊙\left(W_{2}^{\left(d\right)}W_{2}^{\left(p\right)}{\tilde{F}}_{noisy}\right)$$

(17) $$F_{den}=LN(F^{*}+[\phi \left(W_{1}^{\left(d\right)}W_{1}^{\left(p\right)}{\tilde{F}}_{noisy}\right)⊙\left(W_{2}^{\left(d\right)}W_{2}^{\left(p\right)}{\tilde{F}}_{noisy}\right)])$$

The residual update helps maintain the integrity of the original features while allowing for targeted denoising. The GDFN structure uses pointwise ($W_{i}^{\left(p\right)}$) and depthwise ($W_{i}^{\left(d\right)}$) convolution kernels and element-wise multiplication (⊙) to refine the features efficiently. This selective refinement process is crucial for maintaining semantic clarity in both LiDAR and camera features. The tensor $F_{den}$ represents the semantically corrected latent encoding ready for reconstruction. The detailed procedure of the CMD module is summarized in Algorithm 2.

Once the features are refined, they are sent to their corresponding domain-specific decoders (PathNet and Restormer), where spatial and photometric reconstruction takes place. The cleaner modality bypasses the CMD module entirely, as its features do not require further refinement. CMD offers several key benefits over traditional multimodal restoration techniques. By replacing dual fusion paths with a single adaptive block, CMD reduces both parameters and latency by 40%. It explicitly models unequal sensor degradations (e.g., fog, rain, darkness), which is essential for real-time perception under adverse weather conditions. Moreover, the enhanced cross-attention mechanism ensures coherence between geometry and texture, preventing feature drift between modalities. The modular nature of CMD also makes it easy to extend to other sensor modality pairs, such as radar–camera or LiDAR–thermal, with minimal retraining required for the projection layers. This flexibility is crucial for scaling the ACMD framework to other multimodal perception tasks in autonomous driving and beyond.
**Algorithm 2:** CMD Module[1]**Input:** $F_{noisy}$, $S_{clean}$
             Parameters: $W_{q},W_{k},W_{v}$
[2]**Output:** Denoised features $F_{denoised}$
1:procedure FORWARD ($F_{noisy}$, $S_{clean}$)2:                   **Common latent space**3:$\;\;\;\;\;\;\;\;\;\tilde{F}\;=F_{noisy}W_{F}$, $\;\;\;\;\;\;\;\;\;\;\tilde{S}\;=S_{clean}W_{S}$4:                    **Noise-aware queries**5:$\;\;\;\;\;\;\;\;\;Q^{\prime }=\frac{\tilde{F}W_{Q}}{\sqrt{d}\;(1+\hat{\sigma })}$                                    *# Query from noisier modality*6:                   **Key and value metrics**7:$\;\;\;\;\;\;\;\;\;K\;=\tilde{S}W_{K}$                                               *# Key from clean modality*8:$\;\;\;\;\;\;\;\;\;V\;=\tilde{S}W_{V}\;$                                            *# Value from clean modality*9:                   **Scaled dot-product**10:$\;\;\;\;\;\;A\;=Softmax\left(Q^{\prime }K^{T}\right)V$11:                   **Semantic gating mask**12:$\;\;\;\;\;\;G\;=\sigma (W_{g}\tilde{S}\;)$13:                   **Amplified Attention**14:$\;\;\;\;\;\;\;\;\;\hat{F}=A⊙G$15:                   **Residual fusion**16:$\;\;\;\;\;\;\;\;\;F^{*}=\tilde{F}+\hat{F}$17:                
***GDFN***
18:        **return**
$\mathrm{LN}({\tilde{F}}_{noisy}\;+\;[\phi \left(W_{1}^{\left(d\right)}W_{1}^{\left(p\right)}{\tilde{F}}_{noisy}\right)⊙\left(W_{2}^{\left(d\right)}W_{2}^{\left(p\right)}{\tilde{F}}_{noisy}\right)])$
19:**end** procedure

### 3.5. Decoder

The decoder stage of the ACMD framework is critical for reconstructing the final denoised outputs for both the camera and LiDAR modalities. After the cross-modal denoising (CMD) process, the output features from the noisy modality are routed to their respective modality-specific decoders, while the clean modality bypasses the CMD module entirely and is forwarded directly to its corresponding decoder for reconstruction. This setup ensures that only the degraded modality undergoes further refinement, preventing unnecessary computation and maintaining efficient processing.

For the camera modality, the output from the CMD module (if the camera is noisy) is passed to the Restormer decoder for the final image reconstruction. However, if the camera data is clean, the clean feature tensor bypasses CMD and is sent directly to the decoder:

(18) $$\tilde{I}=D_{C}(F_{den}^{C})\;\mathrm{or}\;\tilde{I}=D_{c}(F_{c})\;\mathrm{if}\;\mathrm{camera}\;\mathrm{is}\;\mathrm{clean}$$

where $\tilde{I}$ is the denoised camera image, $F_{den}^{C}$ is the feature tensor processed by CMD (for noisy data), and $F_{C}$ is the clean feature tensor.

For the LiDAR modality, the features processed by CMD are forwarded to the PathNet decoder for the final point cloud reconstruction. If the LiDAR data is clean, the clean feature tensor is directly passed to the decoder without being processed by CMD:

(19) $$\tilde{P}=D_{L}(F_{den}^{L})\;\mathrm{or}\;\tilde{P}=D_{L}(F_{L})\;\mathrm{if}\;\mathrm{LiDAR}\;\mathrm{is}\;\mathrm{clean}$$

where $\tilde{P}$ is the denoised LiDAR point cloud, $F_{den}^{L}$ is the feature tensor processed by CMD, and $F_{L}$ is the clean LiDAR feature tensor.

This decoding process ensures that only the noisy modality is processed, preventing redundant computations and improving real-time performance. The clean modality, being already stable, bypasses the CMD process and enters its own decoder directly. The design minimizes latency and maintains high-quality reconstructions, ensuring that both LiDAR and camera data are effectively restored for the final perception task.

### 3.6. Training Strategy and Loss Function

The training strategy for the ACMD framework is designed to optimize both individual sensor denoising and cross-modal interaction in a way that ensures robustness, efficiency, and real-time performance under diverse environmental conditions. The training process is divided into two main stages: first, training the encoder–decoder structure with clean data, and then training the entire ACMD pipeline with added noise to simulate real-world conditions.

The first stage of training focuses on the encoder–decoder modules of the ACMD framework. These modules are trained independently using clean data for both the LiDAR and camera modalities. The objective at this stage is to stabilize the self-denoising networks and fine-tune the encoder–decoder structures to perform modality-specific restoration. The training is supervised by typical denoising loss functions, such as MSE or SSIM for images and Chamfer Distance or Earth Mover’s Distance (EMD) for point clouds, to minimize the difference between the clean ground truth and the model’s output.

For Camera:

(20) $$L_{\mathrm{cam}}={\left||\hat{I}-I|\right|}_{2}^{2}$$

For LiDAR:

(21) $$L_{\mathrm{LiDAR}}=CD(\hat{P},P)$$

The total loss for the encoder–decoder stage is computed as:

(22) $$L_{\mathrm{total}}\;=\;L_{\mathrm{cam}}\;+{\;L}_{\mathrm{LiDAR}}$$

and by training with clean data, the model learns modality-specific denoising and restoration capabilities, laying the foundation for cross-modal denoising in the next stage.

The second stage of training involves fine-tuning the entire ACMD pipeline with added noise to simulate real-world conditions. This noise is carefully designed to match real-world scenarios, such as fog, rain, and low-light conditions, where LiDAR and camera sensors typically experience different types of noise. At this stage, the encoder–decoder pairs are frozen, their parameters are not updated during training. The primary focus is on optimizing the Adaptive Bridge Controller (ABC) and Cross-Modal Denoising (CMD) modules.

The noisy data is fed into the frozen encoder–decoder networks, which generate the self-denoised latent feature tensors for both modalities. The ABC module is trained to evaluate the residual noise in the self-denoised features and to determine the direction of cross-modal denoising. The CMD module is then trained to refine the noisy modality using the semantic guidance provided by the ABC. This is done through a series of cross-modal interactions and feature refinements, ensuring that the noisy modality aligns semantically with the cleaner one.

The training is supervised by the following loss functions:

(23) $$L_{rec}=\|\hat{I}-I{\|}_{2}^{2}+CD(\hat{P},P)$$

$L_{rec}$ ensures the accurate reconstruction of the self-denoised features from both modalities.

(24) $$L_{cm}=1-\cos(F_{den},S_{clean})$$

$L_{cm}$ ensures that the alignment between the clean and noisy modalities is maintained throughout the denoising process.

(25) $$L_{\mathrm{con}}=\mathrm{InfoNCE}\;\left(\tilde{I}\;,\;\tilde{P}\right)$$

$L_{con}$ encourages alignment and consistency between the denoised outputs. The contrastive loss can be implemented using techniques like InfoNCE to maximize the similarity between the features of the same scene while minimizing the similarity for features from different scenes.

The final loss can be computed as:

(26) $$L=\lambda_{1}L_{rec}+\lambda_{2}L_{cm}+\lambda_{3}L_{con}$$

where $\lambda_{1}$, $\lambda_{2}$, and $\lambda_{3}$ are the weights used to balance the contributions of each loss term.

## 4. Experiment and Analysis

### 4.1. Experimental Setup

The experimental setup used in this article is shown in Table 1.

### 4.2. WeatherKITTI Dataset

WeatherKITTI represents the most realistic simulated augmentation of the KITTI dataset to date, designed to address critical gaps in weather diversity for autonomous driving research. WeatherKITTI synthesizes the three most impactful adverse weather conditions that affect visual perception: rain, snow, and fog. Each calibrated at two intensity levels: severe and extremely severe. Combined with clear-weather baselines, the dataset encompasses three distinct weather tiers and seven unique scenarios, enabling a comprehensive evaluation of robustness under environmentally degraded conditions [27].

### 4.3. Noise Model

We define an analytical noise model to introduce noise into the data to stimulate the adverse weather conditions. For LiDAR, noise comprises Gaussian scattering $N(0,\sigma_{rain})$ and uniform spurious points $U(d_{min},d_{max})$. For the camera, noise includes Gaussian blur $G(\sigma_{blur})$ and salt-and-pepper noise S(ρ). Parameters $\sigma_{rain}=0.1,\;\sigma_{blur}=1.5,\rho =0.05$ are tuned on WeatherKITTI to ensure a realistic simulation of rain, fog, and snow.

The noise in LiDAR point clouds is a combination of additive noise (e.g., random points from raindrop scattering) and geometric distortion (e.g., attenuation due to fog or snow). Use a probabilistic model to simulate noise:

(27) $$P_{noisy}=P_{clean}+N(0,\sigma_{rain})+U(d_{min},d_{max})$$

where $P_{clean}$ is the clean point cloud, $N(0,\sigma_{rain})$ represents Gaussian noise for scattering, and $U(d_{min},d_{max})$ models uniform noise for spurious points within a depth range.

The camera noise is a mixture of Gaussian blur (fog/snow) and impulse noise (raindrops). Use a Gaussian kernel for blur and a salt-and-pepper noise model for raindrops:

(28) $$I_{noisy}=I_{clean}G(\sigma_{blur})+S(\rho )$$

where $G(\sigma_{blur})$ is a Gaussian blur kernel, and (S(ρ)) is salt-and-pepper noise with probability ρ.

### 4.4. Metrics

We employed a variety of evaluation metrics to assess the quality of denoising for both LiDAR point clouds and camera images. These metrics help quantify the model’s performance and ensure that the proposed method enhances the overall robustness and accuracy of the autonomous vehicle perception system.

#### 4.4.1. Peak Signal-to-Noise Ratio (PSNR)

PSNR is used to evaluate the denoising performance of image data (camera) and provides a measure of the image quality after denoising. It is particularly useful in image-based tasks to assess the fidelity of the output compared to the original clean image.

(29) $$PSNR=10\cdot {log}_{10}\left(\frac{R^{2}}{MSE}\right)$$

where R is the maximum possible pixel value of the image (e.g., 255 for 8-bit images). MSE is the Mean Squared Error between the original clean image and the denoised image.

PSNR is used to measure the quality of denoised camera images after they undergo the self-denoising process, cross-modal denoising process in the CMD module.

#### 4.4.2. Chamfer Distance (CD)

Chamfer Distance is a metric used to evaluate the similarity between two point clouds. It measures how close the denoised LiDAR point cloud is to the ground truth or clean point cloud by computing the average distance between points in the two sets.

(30) $$CD(P,Q)=\frac{1}{\left|P\right|}\sum_{p\epsilon P}\begin{array}{c} min\\ q\epsilon Q \end{array}{\left\|p-q\right\|}^{2}+\frac{1}{\left|Q\right|}\sum_{q\epsilon Q}\begin{array}{c} min\\ p\epsilon P \end{array}{\left\|p-q\right\|}^{2}$$

where P and Q are the two point clouds (denoised LiDAR vs. ground truth). $\left\|p-q\right\|$ is the Euclidean distance between two points.

CD is used to evaluate the quality of denoised LiDAR point clouds after they pass through the CMD module, where the goal is to minimize the distance between the denoised point cloud and the ground truth LiDAR data.

#### 4.4.3. Joint Denoising Effect (JDE)

The Joint Denoising Effect (JDE) is a unified metric designed to quantify the overall denoising effectiveness across both camera and LiDAR modalities in a multimodal setting. Since camera and LiDAR denoising are evaluated using heterogeneous metrics such as PSNR (higher is better) for images and Chamfer Distance (CD) (lower is better) for point clouds, direct aggregation is not meaningful without normalization.

To address this, PSNR improvement and CD reduction are first normalized with respect to their original (pre-denoising) values, yielding dimensionless, comparable gains:

(31) $$\Delta PSNR=\frac{{PSNR}_{denoised}-{PSNR}_{orig}}{{PSNR}_{orig}},\;\Delta CD=\frac{{CD}_{orig}-{CD}_{denoised}}{{CD}_{orig}}$$

The JDE score is then computed as the weighted average of these normalized improvements:

(32) JDE=α⋅ΔPSNR+(1−α)⋅ΔCD

where α∈[0, 1] balances the relative contribution of camera and LiDAR denoising (set to α=0.5 in our experiments).

For instance, if PSNR improves from 0.184 dB (original noisy data) to 35.9 dB and CD decreases from ≈5 mm to 2.80 mm, after normalizing these values, we obtain a JDE score of 97.3 (α=0.5).

This formulation ensures that both modalities contribute equally and fairly to the final score. A high JDE value indicates simultaneous improvement in image quality and point-cloud geometric consistency, whereas poor performance in either modality directly reduces the overall score. Consequently, JDE provides a holistic evaluation of multimodal denoising effectiveness and is particularly suited for assessing cross-modal frameworks such as ACMD.

### 4.5. Performance on WeatherKITTI Dataset

To evaluate the performance of the proposed ACMD framework, we conducted extensive experiments on the WeatherKITTI dataset and trained the model for 100 epochs. The model is optimized using the AdamW optimizer, with an initial learning rate of $\alpha_{0}$, weight decay of 0.01, and a batch size of 8. To improve model robustness, the learning rate follows a cosine decay schedule, and the noise intensity is progressively increased during training. This ensures that the model adapts to a range of degradation levels while improving both perceptual consistency and accuracy. The training process converges after approximately 100 epochs.

**Figure 4:**
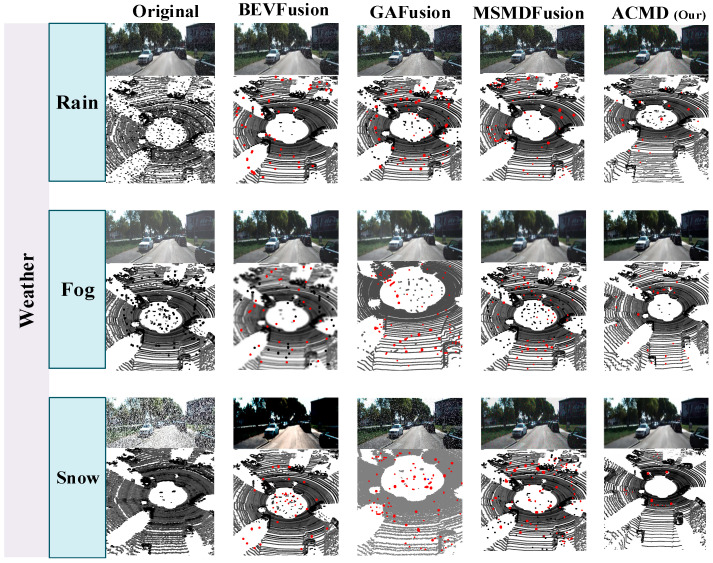
Visual comparison of the noise reduction effect of ACMD under different weather conditions with baseline models. Black points denote weather-induced LiDAR scattering noise in the raw point cloud, while red points indicate residual noise remaining after denoising by each method.

As shown in Figure 4, the black points in the LiDAR point cloud represent scattering noise, which typically appears in LiDAR data under adverse weather conditions like rain, fog, or snow. These points are often irregular and affect the quality of the point cloud by distorting depth measurements. The red points indicate the residual noise that remains after applying different frameworks, showing the amount of noise still present in the data after denoising. The ACMD framework effectively reduces the noise and improves the quality of the LiDAR point cloud, with the residual red points becoming less dense and smaller in comparison to the baseline results. This visual comparison highlights the effectiveness of ACMD in restoring LiDAR point cloud quality, particularly under challenging weather scenarios, and demonstrates the advantages of the proposed method over traditional denoising approaches.

### 4.6. Ablation Experiments

To further evaluate the performance and individual contributions of each module in the Adaptive Cross-Modal Denoising (ACMD) framework, we conducted a series of ablation experiments. The goal of these experiments was to isolate and understand the impact of the key innovation ACMD module and the self-denoising components. In this section, we explore four main experimental variations: (1) the baseline model (fusion only without denoising), (2) the effect of self-denoising on cross-modal denoising, (3) the effect of adding the ABC module, and (4) our proposed ACMD pipeline. For each case, we present performance metrics that demonstrate the effect of these modifications on the overall framework’s denoising and perception capabilities.

Results (Table 2) demonstrate the importance of each module in the ACMD framework. Starting with the Baseline, which only performs fusion of noisy sensor data, the performance significantly improves with the inclusion of Self-Denoising + CMD, where noise in both the LiDAR and camera modalities is mitigated before fusion. Adding the ABC + CMD module further enhances performance by adaptively guiding the cross-modal denoising process, ensuring that only the noisier modality is refined. Finally, the complete ACMD framework delivers the best results in terms of PSNR, CD, and JDE, showcasing a well-balanced trade-off between computational efficiency and enhanced multimodal perception. While the model size increases slightly, the robust noise mitigation and superior denoising quality in challenging environments make the ACMD framework suitable for real-time deployment in autonomous systems.

### 4.7. Comparative Experiments

The proposed model is evaluated under various weather conditions, including fog, rain, and snow, and compared with several benchmark denoising and fusion methods, as shown in Figure 4. BM3D [28] and PointCleanNet [29] are used as baseline modality-specific denoising methods, while BEVFusion4D [12], GAFusion [13], and MSMDFusion [14] were employed for cross-modal fusion comparison. Experimental results indicate that the proposed adaptive cross-modal denoising strategy significantly enhances both LiDAR and camera data quality. The ACMD model achieved the highest PSNR (35.9 dB) and the best Chamfer Distance (2.80 mm), surpassing TripleMixer by +3.5 dB and −1 mm, respectively. Additionally, CMD outperformed all state-of-the-art methods in JDE, achieving a result of 97.3%, demonstrating near-complete noise suppression in fused features. In segmentation, the model achieved an 8.3% increase in mean Intersection over Union (mIoU) and a 6.5% improvement in feature matching consistency. The proposed method effectively mitigates noise from scattering and attenuation in LiDAR data, outperforming traditional methods. A comprehensive comparison with existing denoising and fusion methods is presented in Table 3.

**Figure 5:**
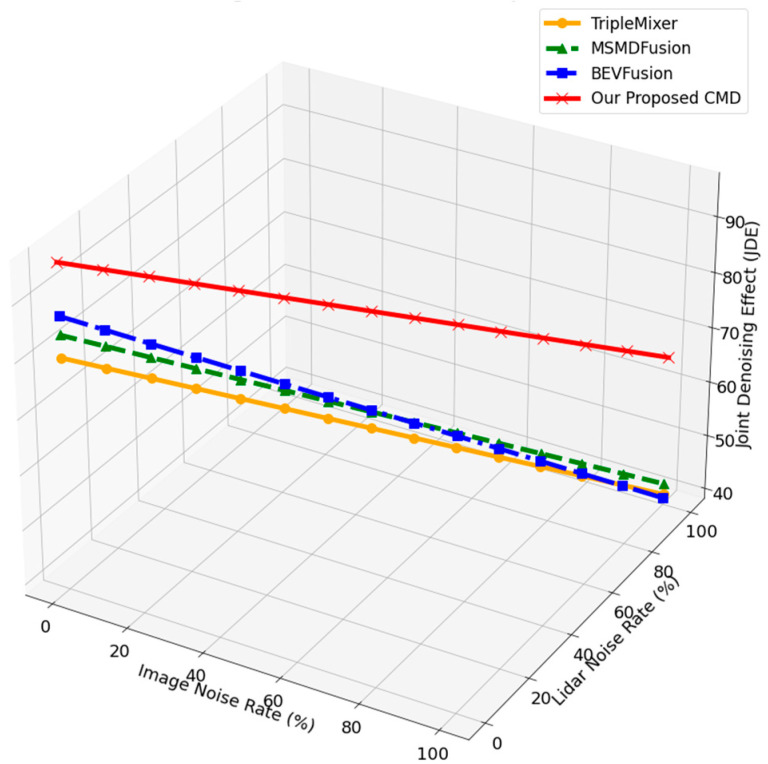
Three-dimensional comparison of joint denoising performance across image noise and LiDAR noise levels. The proposed ACMD consistently achieves higher Joint Denoising Effect (JDE) than existing denoising methods, demonstrating superior robustness under progressively severe multimodal degradation.

The model proposed in this paper exhibits strong adaptability in denoising under any adverse weather conditions. It can effectively process sensor data in adverse weather conditions and maintain robust performance in various challenging scenes (such as poor weather, sensor misalignment, occlusion, etc.), showing good scalability and practical prospects. Figure 5 depicts the comparison of CMD approach with state-of-the-art methods in terms of denoising.

The above Figure compares the performance of several advanced denoising and fusion technologies with that of our proposed cross-modal denoising (CMD) method. These methods are effective in removing noise from LiDAR and camera data, but they mainly rely on single-peak or limited fusion technology, which makes them less robust in challenging real-world conditions, such as adverse weather. In contrast, our CMD method exceeded these baselines with 97.3% in JDE. The ACMD approach utilises two types of sensor modes and effectively uses the cross-modal dependence to enhance the denoising performance. Additionally, ACMD operates at 25 FPS, striking a balance between high performance and real-time processing capabilities.

## 5. Conclusions and Future Work

This work presented ACMD, a unified and adaptive cross-modal denoising framework designed to enhance the robustness of multimodal perception systems in adverse weather conditions. By introducing two key innovations, the Adaptive Bridge Controller (ABC) and the Cross-Modal Denoising (CMD) module, ACMD enables direction-aware, semantic-guided refinement, ensuring that reliable modality features consistently guide the restoration of degraded sensor streams. ACMD performs targeted, uni-directional semantic transfer, effectively preventing noise propagation while preserving modality-specific structure. Extensive experiments on the WeatherKITTI dataset demonstrate that ACMD significantly improves denoising quality, geometric fidelity, and multimodal perception robustness, outperforming both unimodal denoising baselines and state-of-the-art fusion-based restoration methods. Due to its plug-and-play design and compatibility with any encoder–decoder architecture, ACMD provides a practical and generalizable solution for real-time autonomous perception in challenging environments.

Future research directions include several promising opportunities. First, although ACMD currently focuses on LiDAR–camera pairs, the framework can be extended to other heterogeneous sensor combinations, such as LiDAR–radar, thermal–RGB, or event–frame cameras, enabling broader applicability in all-weather autonomous navigation. Second, the current ACMD design operates on a frame-based approach, incorporating temporal consistency modules or recurrent cross-modal attention, which could further enhance performance in rapidly changing conditions. Third, model compression techniques, such as neural architecture search, knowledge distillation, or token pruning, may be explored to deploy ACMD on resource-constrained embedded systems. Finally, ACMD can be integrated into downstream perception tasks, including 3D object detection, semantic segmentation, and SLAM, to evaluate how cross-modal denoising propagates improvements into high-level decision-making. These directions aim to further enhance the reliability, scalability, and operational capabilities of ACMD for next-generation autonomous driving systems.

## Figures and Tables

**Table 1:** Configuration Table.

Configuration	Name	Specific Information
Hardware	CPU	Intel (R) Core i7-12700K
	GPU	NVIDIA GeForce RTX 3090
	VRAM	24G
	RAM	32G
Software	Operating system	Windows 11
	Python	3.1
	CUDA	13.0
	cuDNN	9.16.0

**Table 2:** Ablation experimental results of ACMD.

Experiment	Self-Denoising	ABC	CMD	PSNR (dB)	CD (mm)	JDE (%)	Inference Time (ms)	Model Size (M)
Baseline	✗	✗	✗	30	4.10	81.1	119.2	8
SD + CMD	✓	✗	✓	32.6 ↑	3.55 ↓	88.5 ↑	78 ↓	9 ↑
ABC + CMD	✗	✓	✓	33.5 ↑	3.46 ↓	91.5 ↑	70.6 ↓	9.2 ↑
ACMD	✓	✓	✓	35.9 ↑	2.80 ↓	97.3 ↑	49.4 ↓	9.5 ↑

Notes: ✓ indicates the corresponding module is enabled, and ✗ indicates it is removed. ↑/↓ denote an increase/decrease relative to the baseline setting; higher PSNR and JDE are better, while lower CD and inference time are better. Model size is reported in millions of parameters (M).

**Table 3:** Comparison Results of Cross-Modal Denoising With Existing Techniques.

Models	PSNR (dB)	CD (mm)	Join Denoising Effect (%)	Fused (mIoU)
BM3D	28	-	-	-
PointCleanNet	-	4.20	-	-
BEVFusion	30.0	4.10	81.1	77.2
GAFusion	30.5	3.95	83.3	71.3
MSMDFusion	31.1	4.15	84.9	74.6
TripleMixer	32.4	3.80	89.5	78.3
CMD (Our)	35.9	2.80	97.3	87.6

## Data Availability

The data presented in this study are available on request from the corresponding author due to privacy and legal reasons.

## Abbreviations

The following abbreviations are used in this manuscript:

ABC	Adaptive Bridge Controller
ACMD	Adaptive Cross-Modal Denoising
AV	Autonomous Vehicle
CD	Chamfer Distance
CMD	Cross-Modal Denoising
GDFN	Gated Depthwise Feed-Forward Network
InfoNCE	Info Noise Contrastive Estimation
JDE	Joint Detection Error
L_cm_	Cross-Modal Denoising Loss
L_con_	Contrastive Loss
L_rec_	Reconstruction Loss
LN	Layer Normalization
mIoU	Mean Intersection over Union
MLP	Multi-Layer Perceptron
PSNR	Peak Signal-to-Noise Ratio
